# Progress in understanding the role of the funding agency in the support of evidence-based practice: evaluation results from Canada

**DOI:** 10.1186/1472-6963-14-S2-P77

**Published:** 2014-07-07

**Authors:** Robert McLean, Ian Graham

**Affiliations:** 1 Corporate Strategy and Evaluation Division, International Development Research Centre, Ottawa, ON, Canada; 2 Evaluation Unit, Canadian Institutes of Health Research, Ottawa, ON, Canada; 3 Epidemiology and Community Medicine, University of Ottawa, Ottawa, ON, Canada; 4 Ottawa Hospital Research Institute, Ottawa, ON, Canada

## Background

The Canadian Institutes of Health Research (CIHR) defines knowledge translation (KT) as a dynamic and iterative process that includes the synthesis, dissemination, exchange, and ethically-sound application of knowledge to improve the health of Canadians, provide more effective health services and products, and strengthen the healthcare system. CIHR, the national health research funding agency in Canada, undertook to advance this concept through direct research funding opportunities in KT. Out of a desire to base future programming decisions on evidence, CIHR undertook to critically evaluate the results of this KT funding program.

## Materials and methods

The evaluation employed a method of participatory, utilization-focused evaluation inspired by the principles of integrated KT. It used a mixed methods approach, drawing on both quantitative and qualitative data, and sought participation from funded researchers, knowledge users, as well as other health research funding agencies from around the world. Lines of inquiry included an international organizational scan (n=26), document/data reviews, in-depth qualitative interviews (n=37), targeted on-line surveys (n=379), and case studies (n=5).

## Results

The evaluation uncovered that the KT funding opportunities performed well against typical measures of research success (ie. academic outputs, training objectives, KT outputs), but also, that they performed well against less typical measures such as positioning research for use and actual knowledge application. Data indicate that KT funding opportunities contribute to the fulfillment of the CIHR mandate in a way that is complementary to “investigator-driven” open research. For example, KT funded researchers report that they contribute more often to improving health, strengthening the health care system, and the creation of health services and/or products, whereas open funded researchers report contributing more often to the creation of new health knowledge.

## Conclusions

CIHR’s KT Funding Program has performed well in terms of meeting expected and producing positive unexpected outcomes. Moreover, it has helped to position CIHR for success in an area that is of increasing significance to health research funders across the world. Given that the concept of evidence-based practice and policy is growing in significance, the dissemination of this study (methods and results) will help to fill a gap in knowledge in three areas: the role of a public research funding agency in facilitating KT, the outcomes and impacts KT funding interventions, and how KT can best be evaluated.

